# Socioeconomic deprivation and changes in the retail food environment of Mexico from 2010 to 2020

**DOI:** 10.1016/j.healthplace.2022.102865

**Published:** 2022-08-03

**Authors:** Yenisei Ramírez-Toscano, Carolina Pérez-Ferrer, Usama Bilal, Amy H. Auchincloss, Tonatiuh Barrientos-Gutierrez

**Affiliations:** a Center for Population Health Research, National Institute of Public Health, Avenida Universidad 655, Santa María Ahuacatitlán, Cuernavaca, Morelos, CP 62100, Mexico; b Center for Nutrition and Health Research, National Institute of Public Health, Avenida Universidad 655, Santa María Ahuacatitlán, Cuernavaca, Morelos, CP 62100, Mexico; c National Council for Science and Technology (CONACYT), Av. Insurgentes Sur 1582, Crédito Constructor, Benito Juárez, CP 03940, Ciudad de México, Mexico; d Urban Health Collaborative, Drexel Dornsife School of Public Health, 3600 Market Street, Philadelphia, PA, 19104, USA; e Department of Epidemiology and Biostatistics, Drexel Dornsife School of Public Health, Nesbitt Hall, 3215 Market St, Philadelphia, PA, 19104, USA

**Keywords:** Food environment, Mexico, Latin America, Socioeconomic deprivation

## Abstract

We aimed to analyze the change in the retail food environment of Mexican municipalities from 2010 to 2020, and to assess if these trends were modified by socioeconomic deprivation. We used data from the National Statistical Directory of Economic Units. Changes in the food store count were estimated using fixed-effects Poisson regression models, including coefficients for time, socioeconomic deprivation, and their interaction. We found a rapid growth in convenience stores, seed-grain stores, and supermarkets while small food retail stores declined. Urban areas had a higher count of all types of food stores; however, the steepest increases in food stores were observed in non-urban areas. The increase in convenience stores, supermarkets, specialty food stores, fruit-vegetable stores, and seed-grain stores was greater in the most deprived areas, compared to the least deprived areas. There has been a substantial expansion and rapid change in Mexico’s food environment, mainly driven by increases in convenience stores and supermarkets in more deprived and less urbanized areas.

## 1 Introduction

The retail food environment is the public physical environment that influences people’s food and beverage choices and considers, among other components, the type, availability and accessibility of food stores (Glanz et al., 2005; Ni Mhurchu et al., 2013). Recent epidemiological studies have shown that the retail food environment is an important driver of dietary patterns and that it is linked to obesity and car-diometabolic diseases (Caspi et al., 2012). For instance, studies in Latin America and high-income countries have shown that a lower availability of fresh food stores and higher availability of food stores that sell mainly ultra-processed foods is associated with lower dietary quality, and higher prevalence of obesity and hypertension (Caspi et al., 2012; Cobb et al., 2015; Kaiser et al., 2016; Pérez-Ferrer et al., 2019). While several studies have focused on the potential link between the food retail and health, the dynamics and drivers of food retail remain largely understudied.

The unequal distribution of unhealthy food stores could represent a strong driver for negative dietary and health outcomes, disproportionally affecting residents of low socioeconomic areas (Diez Roux and Mair, 2010). Prior cross-sectional studies suggest that neighborhoods with lower socioeconomic status have higher availability of food stores that promote unhealthy eating, but results have been mixed (Bower et al., 2014; Duran et al., 2013; Franco et al., 2008; Hilmers et al., 2012; Moore and Diez Roux, 2006; Morland et al., 2002; Thornton et al., 2016). For instance, studies in high-income countries reported that compared to neighborhoods of higher socioeconomic status, neighborhoods with lower socioeconomic status have more food stores that promote unhealthy eating (convenience stores in US (Bower et al., 2014) and fast food restaurants in Australia (Thornton et al., 2016)). However, other studies reported more convenience stores and supermarkets in wealthier neighborhoods (Morland et al., 2002). Fewer studies have examined longitudinal changes and those available focused on high-income countries. Overall, the longitudinal evidence has shown that changes in the food environment vary by area-level deprivation (Bilal et al., 2018; Bivoltsis et al., 2019; Filomena et al., 2013; Hobbs et al., 2021; James et al., 2017; Maguire et al., 2015; Pinho et al., 2020; Richardson et al., 2014). Results vary by city context nevertheless, some studies reported that increases in availability of a variety of retail food outlets were most pronounced in low-income neighborhoods (increases in the availability of supermarkets (Bivoltsis et al., 2019; Filomena et al., 2013; Hobbs et al., 2021; Pinho et al., 2020), convenience stores (Pinho et al., 2020), café restaurants (Bivoltsis et al., 2019), takeaway food outlets (Maguire et al., 2015) were largest in the most deprived areas). More evidence is needed for low- and middle-income settings regarding the prevalence and changes over time in socioeconomic disparities in the food environment.

The number and type of retail food stores can determine whether healthy or unhealthy food are available. Large supermarkets and chain convenience stores tend to distribute a large volume of processed and ultra-processed foods rich in sodium, added sugar and fats. In Latin America, traditional specialized markets tend to source unprocessed or minimally processed foods, such as fruits and vegetables, meat, fish and poultry, seeds and legumes, with the exception of smaller stores (called *tiendas de abarrotes* in Mexico), which mainly sell processed and ultra-processed foods (Asfaw, 2008; Clark et al., 2012; Hawkes, 2006, 2008). In areas where self-production of food is uncommon, such as in large cities, the food retail environment determines the dietary choices of a community, representing a key determinant of dietary quality (Popkin and Reardon, 2018).

The transformation towards a more industrialized food environment has been fostered by urbanization, rapid growth in mean per capita income, and liberalization of international trade, including the implementation of the North American Free Trade Agreement (NAFTA) (Friel et al., 2013; Hawkes, 2006; Reardon and Berdegué, 2002). Over the past decades, a rapid growth in supermarkets and convenience stores has been recorded in Latin America (Reardon and Berdegué, 2002; Schwentesius and Gómez, 2002) while small traditional sources of food have stagnated (Hawkes, 2006). However, no study has analyzed how this process is occurring across areas by socioeconomic deprivation in Mexico. Thus, we aimed to analyze the change of food stores in municipalities of Mexico from 2010 to 2020, and to assess if trends are modified by area-level socioeconomic deprivation. We hypothesize that the number of food stores has increased in Mexico from 2010 to 2020, and municipalities with higher socioeconomic deprivation will have a relatively steeper increase in the number of supermarkets, convenience stores and candy-ice cream stores, and a relatively steeper decline in the number of traditional food stores like fruit and vegetable stores, specialty stores and small food retail stores (*tiendas de abarrotes*) compared to those with lower socioeconomic deprivation.

## 2 Methods

### 2.1 Study setting

This study was conducted using data across the 2454 municipalities (*municipios*) updated at 2010 in Mexico. According to the National Census, in 2010 the average population per municipality was 45,763, by 2020 the average population was 51,144 inhabitants per municipality (Instituto Nacional de Estadística Geografía e Informática, 2020a; 2010a). The median area of the municipalities at baseline was 232.9 km^2^, ranging from 85.8 km^2^ to 665.3 km^2^. We used the land area of 2010 since we selected the municipalities that were consistent in boundaries during the study period. Since the economic development and food retail differs by urbanicity, we classified the municipalities into urban and non-urban. We defined urban as municipalities that were part of the *Salud Urbana en América Latina* (SALURBAL) project (Diez Roux et al., 2019), which includes cities with more than 100,000 residents (Quistberg et al., 2019).

### 2.2 Retail food environment data

The main outcome was the number of food stores by type in each municipality and year, obtained from the National Statistical Directory of Economic Units (*DENUE*) for years 2010, 2015 and 2020 conducted by the National Institute of Statistics and Geography of Mexico (*INEGI*). The DENUE is an inventory that contains information on the principal economic activity, size (based on employed personnel), and location of economic units that carry out activities related to manufacturing, commerce, and services. INEGI classified the food stores according to the North American Industrial Classification (NAICS). The information of DENUE is based on the National Economic Censuses, which is the backbone of the National Economic Information Subsystem, representing a direct source of information with a regular (every five years) ground-truthing by government officials (Instituto Nacional de Estadística Geografía e Informática, 2009). The DENUE rounds for 2010, 2015 and 2020 were fully updated after the Economic Censuses of 2009, 2014 and 2019. Detailed information about data collection is available elsewhere (Instituto Nacional de Estadística Geografía e Informática, 2020b).

We categorized food stores in seven mutually exclusive types, based on NAICS codes, product assortment and prior studies (Bridle-Fitzpatrick, 2015; Pérez-Ferrer et al., 2020; Instituto Nacional de Estadística Geografía e Informática, 2018): (1) small food retail stores, (2) chain convenience stores, (3) candy and ice cream stores, (4) specialty food stores (i.e. poultry market, meat market, fish/seafood market, dairy market), (5) fruit and vegetable stores, (6) seed and grain stores, and (7) supermarkets. Chain convenience stores were classified using the name of the store, since NAICS codes do not differentiate between chain convenience and those that are not a franchise. We used the main companies that exist in Mexico: OXXO, 7-Eleven, Circle K, Extra, Bodega Aurrera Express, Super Q, Neto, Pits, Go Mart, Super City, Asturiano, Tiendas 3B, Kiosko, Tent, Chedraui Supercito (de conveniencia, 2021). The definitions, NAICS codes and examples of the food stores categories are available in Supplementary Table S1.

### 2.3 Socioeconomic deprivation

We measured municipality-level socioeconomic deprivation at baseline (2010) using the marginality index. The marginality index is a composite index of area-level socioeconomic deprivation that includes nine variables across four dimensions: access to public services, access to education, and economic and employment conditions. The index was divided into quintiles which correspond to: very low, low, medium, high and very high deprivation (Consejo Nacional de Población, 2010). For the purpose of our analysis, high and very high deprivation were collapsed into one category due to few observations (0.2%) in the very high deprivation category in the non-urban municipalities.

### 2.4 Covariates

We included as a covariate population density measured at the municipality level. Population density was calculated as the total population as reported in the National Census of 2010, 2015 and 2020 (Instituto Nacional de Estadística Geografía e Informática, 2020a; 2015; 2010a), over the total land area of the municipality in km^2^, obtained from INEGI’s Geostatistical framework from 2010 (Instituto Nacional de Estadística Geografía e Informática, 2010b). We selected population density as a time-varying covariate because it is known to be an important factor in food store variation over time and – in this area of research – it is common to adjust for this factor (Pérez-Ferrer et al., 2020; Pinho et al., 2020).

### 2.5 Data analysis

The main objectives of this analysis were to estimate trends in the number of food stores by municipality, and to test whether these trends were modified by socioeconomic deprivation. First, we described area-level characteristics of the municipalities at baseline and for each year of follow-up. We computed medians and measures of dispersion of continuous variables and proportions for categorical variables. Second, we described the outcome variable (number of food stores) by the absolute number of each food store type by year, and the percentage of food stores share.

Third, as the food store data is likely to be zero-inflated, we considered a zero-inflated Poisson regression model, however we decided on fixed-effects Poisson regression model since we wanted to estimate longitudinal change and the fixed-effects approach controls for all time-invariant unmeasured characteristics that vary by municipality. Furthermore, the results focus on changes in food stores as the fixed-effect approach removes municipalities where there was no change across all time periods. Then, to study the change in the number of food stores from 2010 to 2020 we fitted seven separate fixed-effects Poisson regression models, one per type of store, as follows:

$$\text{log}\left(\text{\#}\;food\;stores{\;}_{it}\right)=\beta_{1}*Time+\text{log}\left(Total\;Population{\;}_{it}\right)+\alpha_{i}$$

Where the dependent variable #food stores is the number of each type of food store at municipality *i*, at time *t*, while total population for that municipality and year is the offset; α_i_ is a municipality-specific fixed effect, that controls for time-fixed and municipality-varying confounders; the main exposure variable was time in years since baseline (2010, time of the first DENUE), centered at baseline (2010) and rescaled by dividing it by 10, to allow for a more straightforward interpretation of the regression coefficients. As a result, the exponentiated β_1_ coefficient represents the relative change in food stores per capita per 10-year increment, while controlling for time-invariant municipality-specific unobserved characteristics (Allison, 2005).

Fourth, to test whether trends vary by socioeconomic deprivation, we repeated the models above and added an interaction term between time and the categorical variable for socioeconomic deprivation at baseline, with very low deprivation as the reference category. The model is described in Supplementary Fig. S1. We then calculated linear combinations of coefficients to estimate 10-year changes (and corresponding confidence intervals) for each store type by levels of deprivation. Last, we ran a secondary analysis exploring trends in the proportion of food stores by type, to proxy changes in the composition of the food environment. To do this, we removed the offset for population and replaced it with an offset for the log of the total number of food stores in a municipality. These models included time-varying population density, to control for changes in population.

Because food environments are known to vary by urbanicity (Gómez and Ricketts, 2013), all analyses were stratified by level of urbanization: municipalities that belong to cities of 100,000 or more inhabitants (urban areas), as defined by SALURBAL project and municipalities that are not part of these cities (non-urban areas). All models used robust standard errors clustered at the municipality level. All analyses were conducted in Stata 14 (StataCorp, College Station, TX).

## 3 Results

Table 1 presents characteristics of the 2454 municipalities by urbanization. Most of the municipalities were in non-urban areas (N = 2,048, 83%). Most of the non-urban municipalities were in the South (60.6%) and urban municipalities were concentrated in the Center region (40.4%). Non-urban municipalities had higher levels of socioeconomic deprivation, with 83.7% having medium, high or very high deprivation, as compared to 19.0% for urban municipalities.

Table 2 shows descriptive municipality-level statistics of food stores, for urban and non-urban municipalities. The median count of total food stores per municipality in 2010 was 88, small food retail stores were higher in number than supermarkets, chain convenience stores and seed and grain stores. Overall, the median counts of seed and grain stores, supermarkets and convenience stores were mostly zero, however, compared to non-urban areas, urban areas showed higher median counts of all types of food stores. All municipalities (2,454) had at least one small food retail store in 2010. The percentage of municipalities without specialty food stores, fruit and vegetable stores, and candy and ice cream stores were 14.3%, 25.8% and 23.8% respectively. More than half of municipalities did not have a seed and grain store, convenience store or supermarket.

**Fig. 1:**
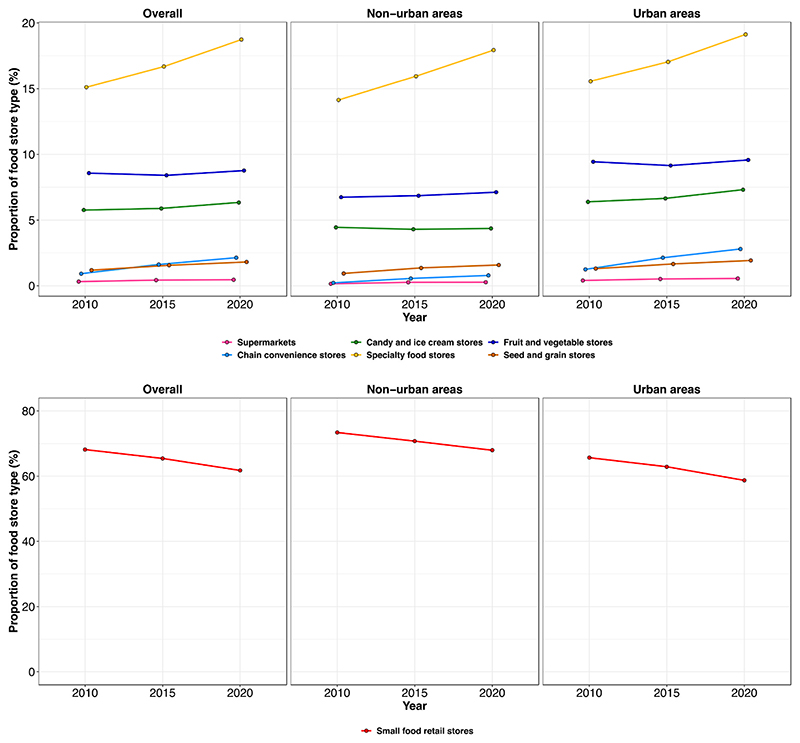
Trends in the proportion of food stores by urbanization in municipalities from Mexico (2010–2020): the National Statistical Directory of Economic Units (DENUE). Proportion of food stores was calculated by dividing the number of each food store type by the total number of food stores.

Fig. 1 shows the proportion of food store types stratified by urbanization and year (2010, 2015, 2020). The bottom panel shows the proportion of small food retail stores, and the top panel shows the proportion of the rest of the food stores. Small food retail stores and specialty food stores dominated the retail food environment, accounting for 68.1% and 15.1% of the total number of stores at baseline, respectively, followed by fruit and vegetable stores, candy and ice cream stores, and seed and grain stores (8.5%, 5.7% and 1.2% respectively). Between 2010 and 2020 there was an 8.5% increase in the total number of food stores. All food stores increased from 2010 to 2020, except for small food retail stores, which decreased slightly. Compared to urban areas, non-urban areas showed a steeper increase in chain convenience stores and supermarkets. In contrast, candy and ice cream stores showed a steeper increase in urban areas (Supplementary Table S2 shows the absolute number of types of food stores). Supplementary Table S3 shows the total number of types of food stores stratified by socioeconomic deprivation. There are more food stores in very low deprivation areas than in high deprivation areas. Within high deprivation areas, a larger share of the stores were small food stores (for example in 2020, 73% within high deprivation areas and 58% within very low deprivation areas).

Table 3 shows the rate ratios for changes in food stores from 2010 to 2020 stratified by urbanization. Similar to the descriptive results, there was an increasing trend for most food store types. Chain convenience stores had the strongest increase, doubling over 10-years (RR = 2.12; 95% CI: 2.00, 2.26). Similarly, the number of food stores increased for seed and grain stores, supermarkets, specialty food stores, and candy and ice cream stores by 50%, 36%, 22% and 8%, respectively. In contrast, small food retail stores declined by 12% (RR = 0.88; 95% CI: 0.87, 0.90). The overall increase across food store types was generally more marked in non-urban areas. Chain convenience stores increased three-fold (RR = 3.21; 95% CI: 2.98, 3.47) in non-urban areas, as compared to a two-fold increase in urban areas (RR = 2.02; 95% CI: 1.90, 2.16). Supermarkets, seed and grain stores, and specialty stores increased by 74%, 70% and 31%, respectively, in non-urban areas, and by 29%, 43%, and 18%, respectively, in urban areas. Small food stores and candy and ice cream stores stayed at similar levels, with steeper changes in urban areas than non-urban areas.

Table 4 shows the rate ratios of change in food stores for each socioeconomic deprivation category stratified by urbanization. Municipalities that had higher deprivation had the largest per-capita increases in chain convenience stores, supermarkets, specialty food stores, and seed and grain stores (p for interaction <0.001). For example, per 10-year increment convenience stores increased 2-fold in municipalities with very low deprivation and 4-fold in municipalities with high or very high deprivation (RR = 1.99; 95% CI: 1.87, 2.12, and RR = 4.45; 95% CI: 3.52, 5.62, respectively). Supermarkets showed a 1.2-fold increase in very low deprivation municipalities and a 2.9-fold increase in high/very high deprivation municipalities (RR = 1.27; 95% CI: 1.20, 1.34, and RR = 2.90; 95% CI: 2.18, 3.85, respectively). In contrast, small food retail stores decreased slightly in less deprived municipalities and increased slightly in the most deprived municipalities (RR = 0.82, 95% CI 0.80, 0.85, and RR = 1.04, 95% CI 1.02, 1.07, respectively). In general, the stronger RR (more rapid growth) observed in more deprived municipalities was similar across urban and non-urban areas, with the exception of convenience stores which had more rapid growth in non-urban areas.

Lastly, the results from secondary analyses that assessed changes in the composition of food stores (proportion of food stores) showed similar patterns to those that represented the change in counts of food stores (Supplementary Table S4 and Supplementary Table S5).

## 4 Discussion

Our findings show that the Mexican food environment continues to be mostly represented by traditional retailers (small food retail stores and specialty food stores). However, small food retail stores had a declining trend, whereas all other types of food stores increased during the 10-year period. The largest relative increase was in chain convenience stores and supermarkets. Changes over time tended to be steepest in non-urban municipalities. Also, municipalities with the highest socioeconomic deprivation had the largest per-capita increases in chain convenience stores, supermarkets, specialty food stores, and seed and grain stores.

The recent trends documented in this study are supported by prior work from Mexico that analyzed periods from the 1990’s and early 2000’s. For example, between 1999 and 2004, chain convenience stores tripled their numbers, reaching 3500 stores nationally (Hawkes, 2006). Chain supermarkets grew from 114 to 561 between 1993 and 2001 (Clark et al., 2012). Recent studies in high-income countries have evaluated trends in the food environment. Similar to our findings, a recent study from the Netherlands showed increases in the counts of convenience stores and decreases in the counts of local food shops (e.g., greengrocers, butchery) from 2004 to 2018 (Pinho et al., 2020). In the US, the evidence follows a similar trend, showing increases in the density of supermarkets, convenience stores, and fast food restaurants, whereas the density of smaller grocery stores decreased (Block et al., 2011; Filomena et al., 2013; James et al., 2017; Richardson et al., 2014). Other studies from Madrid, Australia, New Zeland and UK also found increases in supermarkets (Bilal et al., 2018; Bivoltsis et al., 2019; Hobbs et al., 2021; Maguire et al., 2015), fast food restaurants or take away restaurants (Bivoltsis et al., 2019; Hobbs et al., 2021; Maguire et al., 2015) and convenience stores (Bivoltsis et al., 2019).

This is the first study, to our knowledge, to describe changes in the distribution of the retail food environment by urbanicity and socioeconomic deprivation in Mexico. In general, urban municipalities have access to a higher number of all types of food stores (healthy and unhealthy food outlets). However, the growth of food outlets was somewhat more rapid in non-urban municipalities and in municipalities with higher deprivation. These rapid increases in higher deprivation municipalities were strongest for chain convenience stores and supermarkets, and lowest for food stores that mostly sell unprocessed food (fruit, vegetables, meat, baked products, grains) like specialty stores, fruit/vegetable stores, and seed/grain stores. In summary, the food environment in more deprived municipalities is shifting towards a higher share of stores selling processed and ultra-processed foods, with a declining share of stores selling unprocessed foods.

Although this is the first study that attempts to describe changes in food environment across municipal-level socioeconomic deprivation in Mexico, some longitudinal research in high-income countries have reported similar results. For example, previous research mostly reported that relative to advantaged neighborhoods, socioeconomically disadvantaged neighborhoods experienced a higher increase in convenience stores (in the US (Richardson et al., 2014) and in the Netherlands (Pinho et al., 2020)) and supermarkets (in the Netherlands (Pinho et al., 2020), Australia (Bivoltsis et al., 2019), and US (Filomena et al., 2013)) while travel distance to supermarkets decreased (Hobbs et al., 2021). While most of the existing literature has focused on modern food retailers, a couple of studies have also documented lack of growth or a declining trend in fruit and vegetable stores in lower socioeconomic areas (Bilal et al., 2018).

Our study also showed that small food retailers decreased in the least disadvantaged municipalities and increased in the most disadvantaged areas. In Mexico, low-SES households tend to purchase most of their food and beverages at traditional retailers (Pedraza et al., 2018), which are main sources of less-healthy foods (Bridle-Fitzpatrick, 2015), and sugar-sweetened beverages (Pedraza et al., 2018). Given continued or increased availability of unhealthy foods in high deprivation areas, health disparities in the Mexican population could worsen. While it is possible that implementation of the Mexican tax on sugar-sweetened beverages and non-essential energy dense foods (January 2014) could have reduced consumer demand for small food retail stores in least deprived areas, prior work suggested the tax did not have a large influence on retail operations (Guerrero-López et al., 2017). Rather, we conjecture that losses in small retail stores occurred because the growth of chain stores captured some of the market share of small retailers.

Understanding the distribution of Mexican retail food stores is important to better inform public policies aimed at regulating food environment. Prior work has theorized that changes in the food environment have been driven by the integration of the global food system (Clark et al., 2012; Hawkes, 2006; Swinburn et al., 2011). Starting in the 1980s, Mexico experienced a globalization of consumption, which was greatly accelerated by NAFTA, signed in 1994, leading to increases in investments into Mexican food processing and the growth of multi-national (primarily US) retailers (Clark et al., 2012; Hawkes, 2006). Mexico’s food systems transformations have also been linked with changing ultra-processed foods and beverage markets, and the global trend towards a more highly processed diet (Baker et al., 2020; Marrón-Ponce et al., 2018). The observed exponential growth in food stores have implications for public health, like the parallel changes in patterns of beverages and food consumption in the Mexican population (Pérez-Tepayo et al., 2020; Stern et al., 2014).

The availability of unhealthy food environments can create a supply-side “push” effect on unhealthy diets, which has downstream effects on obesity and chronic diseases (Lake, 2018; Swinburn et al., 2013). The exponential growth of supermarkets and convenience stores that this study found, suggests that policies intervening in the retail food environment should target deprived municipalities and less urbanized areas. Our results provide a view of the distribution and trends of the Mexico’s retail food environment, providing planners and decision-makers insights to adopt actions related to zoning and licensing retailers, that need to be coordinated with already existing policies in Mexico, like stronger restrictions on unhealthy food/beverage marketing, promoting front-of-pack warning labeling, and enhancing the sugar-sweetened beverages tax. Moreover, further studies are needed to better inform the quality of foods sold across all type of food stores.

Our study has some limitations. First, we could not include informal food markets – such as temporary open-air street markets that are open one- or two days per week – because there is no information available about these. Second, we used municipality-level socioeconomic deprivation for 2010 only, this could introduce misclassification if area socioeconomic deprivation changed over time. In this sense, the marginality index used to measure socioeconomic deprivation is a relative measure constructed for an specific census year and categorized in quintiles, therefore it is inadequate for analyzing longitudinal change because it may not be comparable over time. Despite this methodological issue, we did and exploratory analysis to compare municipality-level change on socioeconomic deprivation between 2010 and 2020; results showed that 58% of the municipalities did not change from quantile of deprivation (see Supplementary Table 6). Additionally, we did not evaluate the consumer food environment, which reflects what consumers encounter within stores, like availability, variety, pricing, promotions and nutritional quality of products (Glanz et al., 2005).

## 5 Conclusions

In summary, we found a changing retail food environment in Mexico, especially in non-urban and in the most disadvantaged areas of the country. These findings suggest that distribution and trends differences in food stores may contribute to health inequalities. Tackling these changes in the food environment may represent a strategy to inform public policies aimed at reducing non-communicable diseases and health inequalities, in coordination with already existing policies such as sugar-sweetened beverages and non-essential energy dense food taxes, and front-of pack labeling system.

## Supplementary Material

Supplementary data to this article can be found online at https://doi.org/10.1016/j.healthplace.2022.102865.

Supplementary Material

## Figures and Tables

**Table 1:** Municipality-level characteristics by year (2010, 2015, 2020) and urbanization: the National Statistical Directory of Economic Units (DENUE).

Non-urban areas^a^
	Year
	2010	2015	2020
	*n = 2048*	*n = 2048*	*n = 2048*
Area (km^2^), [median (p25, p75)]	250 (98, 687)		
Population density (residents/ km^2^), [median (p25,p75)]	39 (15, 88)	40 (15, 91)	41 (15, 94)
Total population, [median (p25,p75)]	9793 (3486, 22881)	10211 (3453, 23929)	10379 (3607, 24118)
Geographic region, [%]			
North	18.8		
Center	20.6		
Mexico City	0.0		
South	60.6		
Socioeconomic deprivation, [%]			
Very low	3.5		
Low	12.7		
Medium	42.6		
High and very high	41.1		
**Urban areas** ^a^	*n* = *406*	*n* = *406*	*n* = *406*
Area (km^2^), [median (p25, p75)]	136 (43, 495)		
Population density (residents/ km^2^), [median (p25,p75)]	425 (169, 1161)	459 (192, 1299)	503 (204, 1425)
Total population, [median (p25,p75)]	67472 (21546, 196953)	75909 (23675, 218153)	83265 (25993, 233648)
Geographic region, [%]			
North	16.7		
Center	40.4		
Mexico City	3.9		
South	38.9		
Socioeconomic deprivation, [%]			
Very low	46.8		
Low	34.2		
Medium	17.5		
High and very high	1.5		

p25 = 25th percentile, p75 = 75th percentile.

a Urbanization is defined by the population in 2010: urban areas are municipalities that belong to a city with more than 100,000 residents as defined by SALURBAL (Diez Roux et al., 2019; Quistberg et al., 2019), while non-urban areas refer to all other municipalities (see Methods section).

**Table 2:** Distribution of food store types in municipalities across exam year stratified by urbanization: the National Statistical Directory of Economic Units (DENUE), 2010–2020.

	Counts of food stores, [median (p25,p75)]			Number of municipalities with no food stores, [n (%)]		
Year	2010	2015	2020	2010	2015	2020
**Overall**	***n*** = ***2454***	***n*** = ***2454***	***n*** = ***2454***	***n*** = ***2454***	***n*** = ***2454***	***n*** = ***2454***
Total food stores	88 (30, 269)	92.5 (33, 283)	98 (35, 303)	0 (0.0)	0 (0.0)	0 (0.0)
Small food retail stores	67 (26, 196)	68 (26, 200)	69 (27, 199)	0 (0.0)	1 (0.0)	0 (0.0)
Specialty food stores	11 (2, 38)	14 (3, 46)	17 (4, 55)	350 (14.3)	293 (11.9)	217 (8.8)
Fruit and vegetable stores	4 (0,16)	4 (1,18)	5 (1,20)	634 (25.8)	568 (23.2)	525 (21.4)
Candy and ice cream stores	3 (1, 12)	3 (1, 13)	4 (1, 13)	585 (23.8)	592 (24.1)	565 (23.0)
Seed and grain stores	0 (0, 2)	0 (0, 3)	1 (0, 4)	1449 (59.1)	1258 (51.3)	1157 (47.2)
Chain convenience stores	0 (0, 0)	0 (0, 1)	0 (0, 2)	1974 (80.4)	1672 (68.1)	1527 (62.2)
Supermarkets	0 (0, 0)	0 (0, 0)	0 (0, 1)	2025 (82.5)	1860 (75.8)	1800 (73.4)
**Non-urban areas** ^a^	***n*** = ***2048***	***n*** = ***2048***	***n*** = ***2048***	***n*** = ***2048***	***n*** = ***2048***	***n*** = ***2048***
Total food stores	65 (26, 167.5)	68 (27, 183)	74 (28.5, 190)	0 (0.0)	0 (0.0)	0 (0.0)
Small food retail stores	51 (22, 123)	51 (22, 128)	53 (22, 129.5)	0 (0.0)	1 (0.1)	0 (0.0)
Specialty food stores	7 (1, 24)	10 (2, 29)	11 (3, 34)	349 (17.0)	293 (14.3)	217 (10.6)
Fruit and vegetable stores	2 (0, 9)	3 (0, 10)	3 (0, 12)	629 (30.7)	566 (27.6)	524 (25.6)
Candy and ice cream stores	2 (0, 8)	2 (0, 8)	3 (0, 8)	579 (28.3)	588 (28.7)	562 (27.4)
Seed and grain stores	0 (0, 1)	0 (0, 2)	0 (0, 2)	1355 (66.2)	1203 (58.7)	1117 (54.5)
Chain convenience stores	0 (0, 0)	0 (0, 0)	0 (0, 1)	1823 (89.0)	1590 (77.6)	1460 (71.3)
Supermarkets	0 (0, 0)	0 (0, 0)	0 (0, 0)	1830 (89.4)	1716 (83.8)	1661 (81.1)
**Urban areas** ^a^	***n*** = ***406***	***n*** = ***406***	***n*** = ***406***	***n*** = ***406***	***n*** = ***406***	***n*** = ***406***
Total food stores	623.5 (206, 1840)	674.5 (244, 1922)	739.5 (257, 2060)	0 (0.0)	0 (0.0)	0 (0.0)
Small food retail stores	433 (146, 1231)	459.5 (165, 1240)	476 (177, 1202)	0 (0.0)	0 (0.0)	0 (0.0)
Specialty food stores	89.5 (28, 264)	108 (39, 293)	129.5 (48, 346)	1 (0.3)	0 (0.0)	0 (0.0)
Fruit and vegetable stores	34 (12, 122)	41.5 (14, 139)	48.5 (15, 157)	5 (1.2)	2 (0.5)	1 (0.3)
Candy and ice cream stores	29 (9, 96)	34 (10, 102)	37.5 (12, 117)	6 (1.5)	4 (1.0)	3 (0.7)
Seed and grain stores	3 (1, 18)	6 (2, 23)	8 (2, 30)	94 (23.2)	55 (13.6)	40 (9.9)
Chain convenience stores	2 (0, 15)	6 (1, 33)	8.5 (2, 46)	151 (37.2)	82 (20.2)	67 (16.5)
Supermarkets	1 (0, 7)	1.5 (0, 9)	2 (0, 10)	195 (48.0)	144 (35.5)	139 (34.2)

p25 = 25th percentile, p75 = 75th percentile.

a Urbanization is defined by the population in 2010: urban areas are municipalities that belong to a city with more than 100,000 residents as defined by SALURBAL (Diez Roux et al., 2019; (Quistberg et al., 2019), while non-urban areas refer to all other municipalities (see Methods section).

**Table 3:** Rate ratio of per capita change in food stores per 10-year increase, stratified by urbanization: the National Statistical Directory of Economic Units (DENUE), 2010–2020.

	Overall	Non-urban areas^b^	Urban areas^b^
	Time trend (+10 years)	Time trend (+10 years)	Time trend (+10 years)
	RR (95% CI)	RR (95% CI)	RR (95% CI)
Food store type^a^			
Small food retail stores	0.88 (0.87, 0.90)	0.96 (0.95, 0.97)	0.85 (0.83, 0.87)
Specialty food stores	1.22 (1.20, 1.24)	1.31 (1.29, 1.34)	1.18 (1.16, 1.21)
Fruit and vegetable stores	1.01 (0.98, 1.04)	1.08 (1.04, 1.13)	0.98 (0.95, 1.02)
Candy and ice cream stores	1.08 (1.03, 1.13)	1.02 (0.98, 1.06)	1.10 (1.04, 1.16)
Seed and grain stores	1.50 (1.44, 1.56)	1.70 (1.60, 1.80)	1.43 (1.37, 1.50)
Chain convenience stores	2.12 (2.00, 2.26)	3.21 (2.98, 3.47)	2.02 (1.90, 2.16)
Supermarkets	1.36 (1.30, 1.43)	1.74 (1.60, 1.89)	1.29 (1.22, 1.36)

RR = Rate ratios, 95% CI = 95% Confidence interval.

Overall: Small food retail stores (observations = 7362, municipalities = 2454); Specialty food stores (observations = 6885, municipalities = 2295); F&V stores (observations = 6261, municipalities = 2087); Candy and icre cream (observations = 6297, municipalities = 2099); Seed and grain stores (observations = 4431, municipalities = 1477); Chain convenience stores (observations = 2892, municipalities = 964); Supermarkets (observations = 2043, municipalities = 681).

a Seven fixed-effects Poisson regression models; each outcome (food store type) included an offset with the log of the total population.

b Urbanization is defined by the population in 2010: urban areas are municipalities that belong to a city with more than 100,000 residents as defined by SALURBAL (Diez Roux et al., 2019; Quistberg et al., 2019), while non-urban areas refer to all other municipalities (see Methods section).

**Table 4:** Rate ratio of per capita change in food stores per 10-year increase stratified by socioeconomic deprivation and urbanization: the National Statistical Directory of Economic Units (DENUE), 2010–2020.

	Among very low deprivation	Among low deprivation	Among medium deprivation	Among high and very high deprivation
	RR (95% CI)	RR (95% CI)	RR (95% CI)	RR (95% CI)	p-value^c^
A. Overall					
Food store type^a^					
Change in small food retail stores	0.82 (0.80, 0.85)	0.90 (0.88, 0.92)	0.97 (0.96, 0.99)	1.04 (1.02, 1.07)	< **0.001**
Change in specialty food stores	1.16 (1.13, 1.19)	1.23 (1.20, 1.27)	1.29 (1.26, 1.33)	1.58 (1.50, 1.66)	< **0.001**
Change in fruit and vegetable stores	0.95 (0.91, 1.00)	1.08 (1.04, 1.13)	1.09 (1.03, 1.14)	1.18 (1.08, 1.28)	< **0.001**
Change in candy and ice cream stores	1.11 (1.04, 1.18)	1.06 (0.98, 1.14)	1.03 (0.97, 1.08)	0.95 (0.87, 1.04)	**0.045**
Change in seed and grain stores	1.41 (1.34, 1.48)	1.51 (1.40, 1.64)	1.62 (1.48, 1.77)	2.07 (1.81, 2.36)	< **0.001**
Change in chain convenience stores	1.99 (1.87, 2.12)	2.88 (2.61, 3.18)	3.46 (3.03, 3.95)	4.45 (3.52, 5.62)	< **0.001**
Change in supermarkets	1.27 (1.20, 1.34)	1.63 (1.49, 1.79)	1.83 (1.64, 2.04)	2.90 (2.18, 3.85)	< **0.001**
B. Non-urban areas^b^					
Food store type^a^					
Change in small food retail stores	0.78 (0.74, 0.81)	0.90 (0.88, 0.93)	0.97 (0.96, 0.99)	1.05 (1.02, 1.07)	< **0.001**
Change in specialty food stores	1.09 (1.03, 1.16)	1.22 (1.18, 1.27)	1.29 (1.25, 1.32)	1.59 (1.51, 1.67)	< **0.001**
Change in fruit and vegetable stores	0.90 (0.82, 0.99)	1.05 (0.99, 1.12)	1.07 (1.01, 1.14)	1.17 (1.08, 1.27)	**0.001**
Change in candy and ice cream stores	1.05 (0.93, 1.19)	1.04 (0.97, 1.12)	1.02 (0.97, 1.08)	0.96 (0.88, 1.05)	0.456
Change in seed and grain stores	1.55 (1.21, 1.97)	1.63 (1.48, 1.79)	1.61 (1.47, 1.76)	2.02 (1.79, 2.30)	**0.017**
Change in chain convenience stores	2.64 (2.29, 3.04)	2.99 (2.61, 3.42)	3.84 (3.40, 4.34)	4.79 (3.73, 6.14)	< **0.001**
Change in supermarkets	1.34 (1.15, 1.56)	1.79 (1.54, 2.07)	1.84 (1.63, 2.08)	2.85 (2.14, 3.78)	< **0.001**
C. Urban areas^b^					
Food store type^a^					
Change in small food retail stores	0.83 (0.80, 0.85)	0.90 (0.86, 0.93)	0.99 (0.95, 1.02)	0.92 (0.77, 1.08)	< **0.001**
Change in specialty food stores	1.16 (1.13, 1.19)	1.24 (1.19, 1.29)	1.31 (1.22, 1.41)	1.29 (1.09, 1.53)	**0.002**
Change in fruit and vegetable stores	0.95 (0.91, 1.00)	1.10 (1.04, 1.16)	1.15 (1.02, 1.29)	1.39 (1.03, 1.86)	< **0.001**
Change in candy and ice cream stores	1.11 (1.04, 1.18)	1.07 (0.95, 1.19)	1.04 (0.91, 1.20)	0.78 (0.57, 1.08)	0.198
Change in seed and grain stores	1.41 (1.34, 1.48)	1.47 (1.33, 1.62)	1.66 (1.26, 2.20)	5.19 (1.03, 26.07)	0.243
Change in chain convenience stores	1.97 (1.85, 2.10)	2.83 (2.49, 3.21)	2.48 (1.89, 3.26)	2.47 (1.38, 4.43)	< **0.001**
Change in supermarkets	1.27 (1.19, 1.34)	1.51 (1.35, 1.69)	1.76 (1.40, 2.22)	9.19 (1.14, 73.91)	**0.001**

RR = Rate ratios, 95% CI = 95% Confidence interval. The RRs (estimated coefficients) in this table are from a combination of the coefficients for the main effect of time (year, centered at baseline) and the product of time and each socioeconomic deprivation category (referent category is very low deprivation). Overall: Small food retail stores (observations = 7362, municipalities = 2454); Specialty food stores (observations = 6885, municipalities = 2295); F&V stores (observations = 6261, municipalities = 2087); Candy and icre cream (observations = 6297, municipalities = 2099); Seed and grain stores (observations = 4431, municipalities = 1477); Chain convenience stores (observations = 2892, municipalities = 964); Supermarkets (observations = 2043, municipalities = 681).

a Seven fixed, effects Poisson regression models; each outcome (food store type) included an offset with the log of the total population.

b Urbanization is defined by the population in 2010: urban areas are municipalities that belong to a city with more than 100,000 residents as defined by SALURBAL (Diez Roux et al., 2019; Quistberg et al., 2019), while non-urban areas refer to all other municipalities (see Methods section).

c Interactions between year and municipal-level socioeconomic deprivation were tested for each outcome (food store type). A low p-value suggests that changes in food stores per 10,year increment was different depending on the municipality’s deprivation group.
