# Supplementary material for: Socioeconomic deprivation and changes in the retail food environment of Mexico from 2010 to 2020
Source: Health Place. Author manuscript; Available in PMC 2022 Sep 7. (PMC7613304; doi:10.1016/j.healthplace.2022.102865)
Supplement: Supplementary Material [file EMS152376-supplement-Supplementary_Material.docx]

# SUPPLEMENTARY MATERIAL

**File contents**

**Table S1.** Food store categories.

**Figure S1.** Specification of fixed-effects Poisson regression model with interaction term.

**Table S2.** Counts of food store types and proportion of food stores in municipalities by year (2010, 2015, 2020) and urbanization: the National Statistical Directory of Economic Units (DENUE).

**Table S3.** Counts of food store types in municipalities across socioeconomic deprivation stratified by urbanization and year (2010 and 2020): the National Statistical Directory of Economic Units (DENUE).

**Table S4.** Adjusted rate ratio of per capita change in proportion of food stores per 10-year increase, stratified by urbanization: the National Statistical Directory of Economic Units (DENUE), 2010-2020.

**Table S5.** Adjusted rate ratio of per capita change in proportion of food stores per 10-year increase stratified by socioeconomic deprivation and urbanization: the National Statistical Directory of Economic Units (DENUE), 2010-2020.

**Table S6.** Change in the socioeconomic deprivation at municipality level, 2010 and 2020.

| **Food store type** | **NAICS codes of the economic unit** | **Examples of food stores** |
| --- | --- | --- |
| Small food retail stores (*abarrotes*) | 461110 - Retail trade in grocery stores + 461213 - Retail trade of nonalcoholic beverages and ice + 462112 - Retail trade in minimarkets minus ‘chain convenience stores’ | Traditional stores that sell milk, cheese, cream, cold cuts, sweets, cookies, bread, cakes, snacks, fried foods, preserves, canned goods, bottled purified water, soft drinks, juices and nectars, hydrating drinks, energy drinks, beer, packaged wines and spirits, cigars, egg, toilet paper, detergent, soap, paper napkins, disposable kitchen utensils. No chain names available. |
| Chain convenience stores | Searched by name because NAICS does not differentiate between the chain convenience stores and those local stores that are similar. Names searched: OXXO, 7-Eleven, Circle K, Extra, Bodega Aurrera Express, Super Q, Neto, Pits, Go Mart, Super City, Asturiano, Tiendas 3B, Kiosko, Tent, Chedraui Supercito | OXXO, 7-Eleven, Circle K, Extra, Bodega Aurrera Express, Super Q, Neto, Pits, Go Mart, Super City, Asturiano, Tiendas 3B, Kiosko, Tent, Chedraui Supercito. |
| Candy and ice cream stores | 461160 - Retail trade of candies and confectionery raw materials + 461170 - Retail trade of popsicles and ice cream | Local stores that sell candies (sweets, chocolates, chewing gum, bonbons, regional sweets, gelatin powders, flavorings and colorants, and other raw materials used in the manufacture of confectionery products), and ice cream and popsicles. No chain names available. |
| Specialty food stores | 461121 - Retail trade of red meat + 461122 - Retail trade of poultry + 461123 - Retail trade of fish and seafood + 461150 - Retail trade of milk, other dairy products and sausages + 461190 - Retail trade of other food (coffee, bread, bakery, tortillas, eggs) | Traditional stores that sell red meat (raw or semi-cooked beef, pork, lamb, goat and other species of red meat animals), poultry (poultry offal, chicken, quail, duck, turkey), fishmongers (fish or shellfish fresh, dried, salted and frozen, and other marine products), dairy and sausages (milk, cream, butter, yogurt, cheese, chorizo, sausage, mortadella, ham, pork cheese), and other food (coffee, bread, bakery, tortillas, eggs). These stores could be inside public food markets or not. No chain names available. |
| Fruit and vegetable stores | 461130 - Retail trade of fresh fruits and vegetables | Traditional stores mainly dedicated to the specialized retail trade of fresh fruits and vegetables. These stores could be inside public food markets or not. No chain names available. |
| Seed and grain stores | 461140 - Retail trade of seeds and grain, spices and dried chili | Traditional stores that sell seeds and food grains, spices (cloves, pepper, saffron, cumin, nutmeg, cinnamon) and dried chili peppers. These stores could be inside public food markets or not. No chain names available. |
| Supermarkets | 462111 - Retail trade in supermarkets minus ‘chain convenience stores’ | Wal-Mart, H-E-B, Soriana, Comercial Mexicana, Superama, City-Market, Fresko, Costco, Sam`s Club, among others. |
| Source: Own elaboration adapted from NAICS 2018. The National Institute of Statistics and Geography (INEGI). North American Industry Classification System (NAICS) 2018. Mexico; 2018. | | |

## **Table S1. Food store categories.**


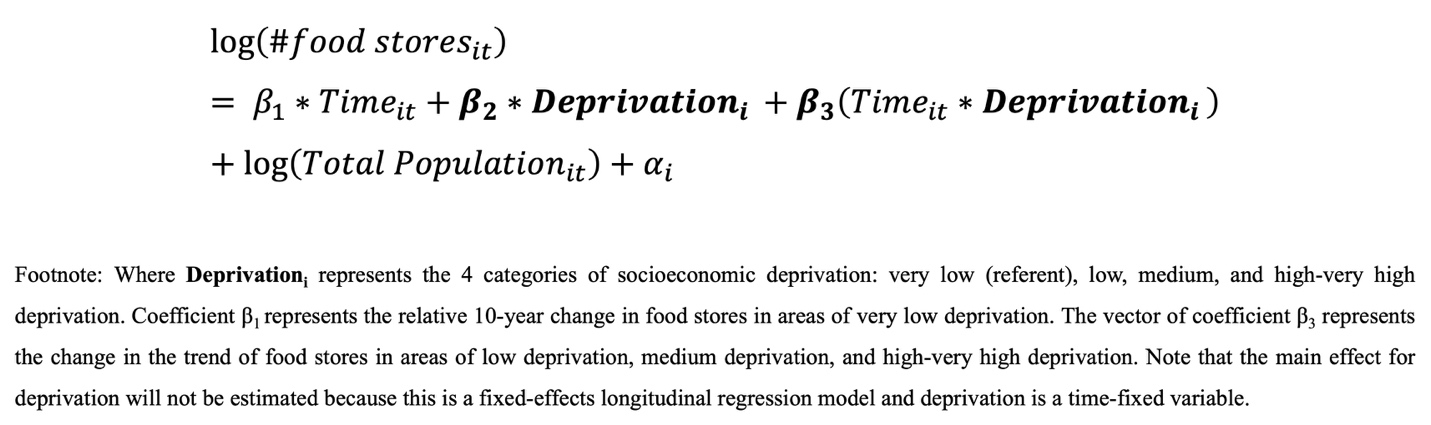


**Figure S1. Specification of fixed-effects Poisson regression model with interaction term.**

|  | **Overall** | | |  | **Non-urban areas*** | | |  | **Urban areas*** | | |
| --- | --- | --- | --- | --- | --- | --- | --- | --- | --- | --- | --- |
| **Year** | **2010** | **2015** | **2020** |  | **2010** | **2015** | **2020** |  | **2010** | **2015** | **2020** |
| **Number of municipalities** | ***2,454*** | ***2,454*** | ***2,454*** |  | ***2,048*** | ***2,048*** | ***2,048*** |  | ***406*** | ***406*** | ***406*** |
| **Counts of food store types (% of total)** | | |  |  |  |  |  |  |  |  |  |
| **Total** | 977,393 | 1,020,936 | 1,060,741 |  | 313,115 | 330,512 | 348,778 |  | 664,278 | 690,424 | 711,963 |
|  |  |  |  |  |  |  |  |  |  |  |  |
| **Small food retail stores** | 666,008 | 667,867 | 655,071 |  | 229,786 | 233,802 | 237,007 |  | 436,222 | 434,065 | 418,064 |
| **(% of total)** | 68.1% | 65.4% | 61.8% |  | 73.4% | 70.7% | 68.0% |  | 65.7% | 62.9% | 58.7% |
|  |  |  |  |  |  |  |  |  |  |  |  |
| **Specialty food stores** | 147,622 | 170,343 | 198,748 |  | 44,254 | 52,697 | 62,557 |  | 103,368 | 117,646 | 136,191 |
| **(% of total)** | 15.1% | 16.7% | 18.7% |  | 14.1% | 15.9% | 17.9% |  | 15.6% | 17.0% | 19.1% |
|  |  |  |  |  |  |  |  |  |  |  |  |
| **Fruit and vegetable stores** | 83,789 | 85,810 | 93,007 |  | 21,098 | 22,656 | 24,835 |  | 62,691 | 63,154 | 68,172 |
| **(% of total)** | 8.6% | 8.4% | 8.8% |  | 6.7% | 6.9% | 7.1% |  | 9.4% | 9.1% | 9.6% |
|  |  |  |  |  |  |  |  |  |  |  |  |
| **Candy and ice cream stores** | 56,320 | 60,088 | 67,232 |  | 13,897 | 14,188 | 15,200 |  | 42,423 | 45,900 | 52,032 |
| **(% of total)** | 5.8% | 5.9% | 6.3% |  | 4.4% | 4.3% | 4.4% |  | 6.4% | 6.6% | 7.3% |
|  |  |  |  |  |  |  |  |  |  |  |  |
| **Seed and grain stores** | 11,581 | 15,909 | 19,192 |  | 2,926 | 4,482 | 5,498 |  | 8,655 | 11,427 | 13,694 |
| **(% of total)** | 1.2% | 1.6% | 1.8% |  | 0.9% | 1.4% | 1.6% |  | 1.3% | 1.7% | 1.9% |
|  |  |  |  |  |  |  |  |  |  |  |  |
| **Chain convenience stores** | 8,953 | 16,521 | 22,637 |  | 687 | 1,822 | 2,747 |  | 8,266 | 14,699 | 19,890 |
| **(% of total)** | 0.9% | 1.6% | 2.1% |  | 0.2% | 0.6% | 0.8% |  | 1.2% | 2.1% | 2.8% |
|  |  |  |  |  |  |  |  |  |  |  |  |
| **Supermarkets** | 3,120 | 4,398 | 4,854 |  | 467 | 865 | 934 |  | 2,653 | 3,533 | 3,920 |
| **(% of total)** | 0.3% | 0.4% | 0.5% |  | 0.1% | 0.3% | 0.3% |  | 0.4% | 0.5% | 0.6% |
| * Urbanization is defined by the population in 2010: urban areas are municipalities that belong to a city with more than 100,000 residents as defined by SALURBAL (Diez Roux et al., 2019; Quistberg et al., 2019), while non-urban areas refer to all other municipalities (see Methods section). | | | | | | | | | | | |

**Table S2. Counts of food store types and proportion of food stores in municipalities by year (2010, 2015, 2020) and urbanization: the National Statistical Directory of Economic Units (DENUE).**

|  | **Overall** | | | |  | **Non-urban areas*** | | | |  | **Urban areas*** | | | |
| --- | --- | --- | --- | --- | --- | --- | --- | --- | --- | --- | --- | --- | --- | --- |
| **Socioeconomic deprivation** | Very low | Low | Medium | High and very high |  | Very low | Low | Medium | High and very high |  | Very low | Low | Medium | High and very high |
| **Number of municipalities** | n= 262 | n= 400 | n= 944 | n= 848 |  | n= 72 | n= 261 | n= 873 | n= 842 |  | n= 190 | n= 139 | n= 71 | n= 6 |
| **2010** |  |  |  |  |  |  |  |  |  |  |  |  |  |  |
| ***Counts of food store types (% of total)*** | |  |  |  |  |  |  |  |  |  |  |  |  |  |
| **Total** | 545,028 | 172,875 | 189,136 | 70,354 |  | 22,783 | 62,626 | 159,098 | 68,608 |  | 522,245 | 110,249 | 30,038 | 1,746 |
|  |  |  |  |  |  |  |  |  |  |  |  |  |  |  |
| **Small food retail stores** | 355,778 | 120,699 | 134,393 | 55,138 |  | 17,769 | 45,167 | 112,995 | 53,855 |  | 338,009 | 75,532 | 21,398 | 1,283 |
|  | 65.3% | 69.8% | 71.1% | 78.4% |  | 78.0% | 72.1% | 71.0% | 78.5% |  | 64.7% | 68.5% | 71.2% | 73.5% |
| **Specialty food stores** | 83,803 | 26,546 | 29,311 | 7,962 |  | 2,546 | 9,283 | 24,727 | 7,698 |  | 81,257 | 17,263 | 4,584 | 264 |
|  | 15.4% | 15.4% | 15.5% | 11.3% |  | 11.2% | 14.8% | 15.5% | 11.2% |  | 15.6% | 15.7% | 15.3% | 15.1% |
| **Fruit and vegetable stores** | 51,746 | 13,306 | 14,551 | 4,186 |  | 906 | 3,983 | 12,143 | 4,066 |  | 50,840 | 9,323 | 2,408 | 120 |
|  | 9.5% | 7.7% | 7.7% | 5.9% |  | 4.0% | 6.4% | 7.6% | 5.9% |  | 9.7% | 8.5% | 8.0% | 6.9% |
| **Candy and ice cream stores** | 36,138 | 9,355 | 8,412 | 2,415 |  | 1,061 | 3,253 | 7,234 | 2,349 |  | 35,077 | 6,102 | 1,178 | 66 |
|  | 6.6% | 5.4% | 4.4% | 3.4% |  | 4.7% | 5.2% | 4.5% | 3.4% |  | 6.7% | 5.5% | 3.9% | 3.8% |
| **Seed and grain stores** | 6,981 | 2,026 | 1,961 | 613 |  | 137 | 575 | 1,610 | 604 |  | 6,844 | 1,451 | 351 | 9 |
|  | 1.3% | 1.2% | 1.0% | 0.9% |  | 0.6% | 0.9% | 1.0% | 0.9% |  | 1.3% | 1.3% | 1.2% | 0.5% |
| **Chain convenience stores** | 8,014 | 616 | 300 | 23 |  | 235 | 224 | 209 | 19 |  | 7,779 | 392 | 91 | 4 |
|  | 1.5% | 0.4% | 0.2% | 0.0% |  | 1.0% | 0.4% | 0.1% | 0.0% |  | 1.5% | 0.4% | 0.3% | 0.2% |
| **Supermarkets** | 2,568 | 327 | 208 | 17 |  | 129 | 141 | 180 | 17 |  | 2,439 | 186 | 28 | 0 |
|  | 0.5% | 0.2% | 0.1% | 0.0% |  | 0.6% | 0.2% | 0.1% | 0.0% |  | 0.5% | 0.2% | 0.1% | - |
|  |  |  |  |  |  |  |  |  |  |  |  |  |  |  |
| **2020** |  |  |  |  |  |  |  |  |  |  |  |  |  |  |
| ***Counts of food store types (% of total)*** | | |  |  |  |  |  |  |  |  |  |  |  |  |
| **Total** | 569,375 | 191,778 | 214,691 | 84,897 |  | 22,254 | 65,455 | 178,408 | 82,661 |  | 547,121 | 126,323 | 36,283 | 2,236 |
|  |  |  |  |  |  |  |  |  |  |  |  |  |  |  |
| **Small food retail stores** | 330,036 | 121,312 | 141,774 | 61,949 |  | 15,655 | 43,104 | 117,787 | 60,461 |  | 314,381 | 78,208 | 23,987 | 1,488 |
|  | 58.0% | 63.3% | 66.0% | 73.0% |  | 70.3% | 65.9% | 66.0% | 73.1% |  | 57.5% | 61.9% | 66.1% | 66.5% |
| **Specialty food stores** | 107,517 | 36,783 | 40,997 | 13,451 |  | 3,137 | 12,145 | 34,240 | 13,035 |  | 104,380 | 24,638 | 6,757 | 416 |
|  | 18.9% | 19.2% | 19.1% | 15.8% |  | 14.1% | 18.6% | 19.2% | 15.8% |  | 19.1% | 19.5% | 18.6% | 18.6% |
| **Fruit and vegetable stores** | 54,063 | 16,215 | 17,358 | 5,371 |  | 931 | 4,514 | 14,218 | 5,172 |  | 53,132 | 11,701 | 3,140 | 199 |
|  | 9.5% | 8.5% | 8.1% | 6.3% |  | 4.2% | 6.9% | 8.0% | 6.3% |  | 9.7% | 9.3% | 8.7% | 8.9% |
| **Candy and ice cream stores** | 44,342 | 11,126 | 9,303 | 2,461 |  | 1,280 | 3,618 | 7,905 | 2,397 |  | 43,062 | 7,508 | 1,398 | 64 |
|  | 7.8% | 5.8% | 4.3% | 2.9% |  | 5.8% | 5.5% | 4.4% | 2.9% |  | 7.9% | 5.9% | 3.9% | 2.9% |
| **Seed and grain stores** | 10,751 | 3,476 | 3,534 | 1,431 |  | 239 | 1,018 | 2,862 | 1,379 |  | 10,512 | 2,458 | 672 | 52 |
|  | 1.9% | 1.8% | 1.6% | 1.7% |  | 1.1% | 1.6% | 1.6% | 1.7% |  | 1.9% | 1.9% | 1.9% | 2.3% |
| **Chain convenience stores** | 18,923 | 2,262 | 1,290 | 162 |  | 805 | 777 | 1,018 | 147 |  | 18,118 | 1,485 | 272 | 15 |
|  | 3.3% | 1.2% | 0.6% | 0.2% |  | 3.6% | 1.2% | 0.6% | 0.2% |  | 3.3% | 1.2% | 0.7% | 0.7% |
| **Supermarkets** | 3,743 | 604 | 435 | 72 |  | 207 | 279 | 378 | 70 |  | 3,536 | 325 | 57 | 2 |
|  | 0.7% | 0.3% | 0.2% | 0.1% |  | 0.9% | 0.4% | 0.2% | 0.1% |  | 0.6% | 0.3% | 0.2% | 0.1% |
| * Urbanization is defined by the population in 2010: urban areas are municipalities that belong to a city with more than 100,000 residents as defined by SALURBAL (Diez Roux et al., 2019; Quistberg et al., 2019), while non-urban areas refer to all other municipalities (see Methods section). | | | | | | | | | | | | | | |

**Table S3. Counts of food store types in municipalities across socioeconomic deprivation stratified by urbanization and year (2010 and 2020): the National Statistical Directory of Economic Units (DENUE).**

|  | **Overall** |  | **Non-urban areas*** |  | **Urban areas*** |
| --- | --- | --- | --- | --- | --- |
|  | **Time trend (+10 years)** |  | **Time trend (+10 years)** |  | **Time trend (+10 years)** |
|  | **RR (95% CI)** |  | **RR (95% CI)** |  | **RR (95% CI)** |
| ***Food store type^1^*** |  |  |  |  |  |
| **Small food retail stores** | 0.91 (0.90, 0.91) |  | 0.93 (0.92, 0.93) |  | 0.90 (0.89, 0.90) |
| **Specialty food stores** | 1.23 (1.22, 1.25) |  | 1.27 (1.25, 1.29) |  | 1.21 (1.20, 1.23) |
| **Fruit and vegetable stores** | 1.02 (1.00, 1.05) |  | 1.04 (1.01, 1.07) |  | 1.02 (0.98, 1.05) |
| **Candy and ice cream stores** | 1.10 (1.06, 1.15) |  | 0.98 (0.95, 1.02) |  | 1.14 (1.09, 1.20) |
| **Seed and grain stores** | 1.51 (1.45, 1.58) |  | 1.65 (1.56, 1.74) |  | 1.46 (1.39, 1.54) |
| **Chain convenience stores** | 2.41 (2.28, 2.54) |  | 3.36 (3.10, 3.63) |  | 2.30 (2.19, 2.43) |
| **Supermarkets** | 1.51 (1.43, 1.60) |  | 1.75 (1.60, 1.92) |  | 1.45 (1.35, 1.55) |
| ^1^ Seven fixed-effects Poisson regression models adjusted for population density; each outcome (food store type) included an offset with the log of the total of food stores. | | | | | |
| * Urbanization is defined by the population in 2010: urban areas are municipalities that belong to a city with more than 100,000 residents as defined by SALURBAL (Diez Roux et al., 2019; Quistberg et al., 2019), while non-urban areas refer to all other municipalities (see Methods section). | | | | | |
| RR = Rate ratios, 95% CI = 95% Confidence interval. | | | | | |
| Overall: Small food retail stores (observations=7362, municipalities=2454); Specialty food stores (observations=6885, municipalities=2295); F&V stores (observations=6261, municipalities=2087); Candy and ice cream (observations=6297, municipalities=2099); Seed and grain stores (observations=4431, municipalities=1477);Chain convenience stores (observations=2892, municipalities=964); Supermarkets (observations=2043, municipalities=681). | | | | | |

**Table S4. Adjusted rate ratio of per capita change in proportion of food stores per 10-year increase, stratified by urbanization: the National Statistical Directory of Economic Units (DENUE), 2010-2020.**

|  | **Among very low deprivation** | **Among low deprivation** | **Among medium deprivation** | **Among high and very high deprivation** |  |
| --- | --- | --- | --- | --- | --- |
|  | **RR (95% CI)** | **RR (95% CI)** | **RR (95% CI)** | **RR (95% CI)** | **p-value^a^** |
| **A. Overall** |  |  |  |  |  |
| **Food store type^1^** |  |  |  |  |  |
| **Change in small food retail stores** | 0.89 (0.88, 0.90) | 0.91 (0.91, 0.92) | 0.93 (0.92, 0.94) | 0.93 (0.92, 0.94) | **<0.001** |
| **Change in specialty food stores** | 1.21 (1.19, 1.23) | 1.23 (1.21, 1.26) | 1.23 (1.21, 1.25) | 1.41 (1.36, 1.46) | **<0.001** |
| **Change in fruit and vegetable stores** | 1.00 (0.96, 1.04) | 1.09 (1.04, 1.13) | 1.04 (1.00, 1.08) | 1.05 (0.99, 1.12) | **0.036** |
| **Change in candy and ice cream stores** | 1.17 (1.11, 1.23) | 1.04 (0.98, 1.11) | 0.98 (0.93, 1.02) | 0.85 (0.79, 0.92) | **<0.001** |
| **Change in seed and grain stores** | 1.46 (1.38, 1.55) | 1.52 (1.41, 1.64) | 1.54 (1.42, 1.67) | 1.86 (1.65, 2.09) | **0.005** |
| **Change in chain convenience stores** | 2.29 (2.17, 2.42) | 3.03 (2.79, 3.29) | 3.26 (2.86, 3.72) | 4.15 (3.30, 5.21) | **<0.001** |
| **Change in supermarkets** | 1.44 (1.34, 1.55) | 1.70 (1.54, 1.87) | 1.75 (1.56, 1.97) | 2.75 (2.08, 3.64) | **<0.001** |
| **B. Non-urban areas*** |  |  |  |  |  |
| **Food store type^1^** |  |  |  |  |  |
| **Change in small food retail stores** | 0.91 (0.89, 0.92) | 0.92 (0.91, 0.93) | 0.93 (0.93, 0.94) | 0.93 (0.92, 0.94) | **0.008** |
| **Change in specialty food stores** | 1.24 (1.19, 1.30) | 1.23 (1.20, 1.27) | 1.24 (1.21, 1.26) | 1.42 (1.37, 1.47) | **<0.001** |
| **Change in fruit and vegetable stores** | 1.02 (0.94, 1.10) | 1.06 (1.01, 1.11) | 1.03 (0.98, 1.08) | 1.04 (0.98, 1.12) | 0.852 |
| **Change in candy and ice cream stores** | 1.22 (1.07, 1.39) | 1.05 (0.98, 1.11) | 0.97 (0.92, 1.02) | 0.85 (0.79, 0.92) | **<0.001** |
| **Change in seed and grain stores** | 1.80 (1.41, 2.30) | 1.67 (1.51, 1.84) | 1.55 (1.43, 1.68) | 1.83 (1.64, 2.04) | 0.100 |
| **Change in chain convenience stores** | 3.18 (2.72, 3.72) | 3.09 (2.72, 3.52) | 3.62 (3.18, 4.12) | 4.44 (3.47, 5.69) | **0.042** |
| **Change in supermarkets** | 1.56 (1.32, 1.84) | 1.81 (1.55, 2.12) | 1.72 (1.50, 1.98) | 2.64 (1.99, 3.49) | **0.014** |
| **C. Urban areas*** |  |  |  |  |  |
| **Food store type^1^** |  |  |  |  |  |
| **Change in small food retail stores** | 0.89 (0.88, 0.90) | 0.91 (0.90, 0.92) | 0.93 (0.91, 0.95) | 0.90 (0.87, 0.94) | **0.001** |
| **Change in specialty food stores** | 1.21 (1.19, 1.23) | 1.23 (1.20, 1.27) | 1.23 (1.17, 1.30) | 1.25 (1.12, 1.39) | 0.689 |
| **Change in fruit and vegetable stores** | 1.00 (0.96, 1.04) | 1.10 (1.04, 1.16) | 1.07 (0.96, 1.20) | 1.30 (1.12, 1.51) | **0.001** |
| **Change in candy and ice cream stores** | 1.16 (1.10, 1.23) | 1.04 (0.94, 1.14) | 0.97 (0.86, 1.10) | 0.77 (0.49, 1.22) | **0.003** |
| **Change in seed and grain stores** | 1.45 (1.37, 1.54) | 1.46 (1.34, 1.60) | 1.58 (1.19, 2.09) | 4.98 (1.16, 21.43) | 0.387 |
| **Change in chain convenience stores** | 2.26 (2.14, 2.39) | 3.00 (2.70, 3.33) | 2.35 (1.82, 3.03) | 2.42 (1.71, 3.44) | **<0.001** |
| **Change in supermarkets** | 1.43 (1.33, 1.55) | 1.59 (1.40, 1.79) | 1.66 (1.32, 2.09) | 9.29 (1.54, 56.04) | 0.073 |
| ^1^ Seven fixed,effects Poisson regression models adjusted for population density; each outcome (food store type) included an offset with the log of the total of food stores | | | | | |
| * Urbanization is defined by the population in 2010: urban areas are municipalities that belong to a city with more than 100,000 residents as defined by SALURBAL (Diez Roux et al., 2019; Quistberg et al., 2019), while non-urban areas refer to all other municipalities (see Methods section). | | | | | |
| ^a^ Interactions between year and municipal,level socioeconomic deprivation were tested for each outcome (food store type). A low p-value suggests that changes in food stores per 10,year increment was different depending on the municipality’s deprivation group. | | | | | |
| RR = Rate ratios, 95% CI = 95% Confidence interval. The RRs (estimated coefficients) in this table are from a combination of the coefficients for the main effect of time (year, centered at baseline) and the product of time and each socioeconomic deprivation category (referent category is very low deprivation). | | | | | |
| Overall: Small food retail stores (observations=7362, municipalities=2454); Specialty food stores (observations=6885, municipalities=2295); F&V stores (observations=6261, municipalities=2087); Candy and ice cream (observations=6297, municipalities=2099); Seed and grain stores (observations=4431, municipalities=1477);Chain convenience stores (observations=2892, municipalities=964); Supermarkets (observations=2043, municipalities=681). | | | | | |

## **Table S5. Adjusted rate ratio of per capita change in proportion of food stores per 10-year increase stratified by socioeconomic deprivation and urbanization: the National Statistical Directory of Economic Units (DENUE), 2010-2020.**

|  | **Number of municipalities, [n (%)]** |
| --- | --- |
| **Socioeconomic deprivation, 2010 and 2020** |  |
| No change | 1426 (58.1) |
| Consistently very low | 262 |
| Consistently low | 69 |
| Consistently medium | 368 |
| Consistently high and very high | 727 |
|  |  |
| Increasing deprivation 1 degree | 58 (2.4) |
| Medium to high/very high | 58 |
|  |  |
| Decreasing deprivation 1 degree | 908 (37.0) |
| Low to very low | 331 |
| Medium to low | 457 |
| High/very high to medium | 120 |
|  |  |
| Decreasing deprivation 2 degrees | 62 (2.5) |
| Medium to very low | 61 |
| High/very high to low | 1 |

**Table S6. ﻿Change in the socioeconomic deprivation at municipality level, 2010 and 2020.**

# References for supplementary material

Diez Roux, A. V., Slesinski, S.C., Alazraqui, M., Caiaffa, W.T., Frenz, P., Jordán Fuchs, R., Miranda, J.J., Rodriguez, D.A., Dueñas, O.L.S., Siri, J., Vergara, A.V., 2019. A Novel International Partnership for Actionable Evidence on Urban Health in Latin America: LAC‐Urban Health and SALURBAL. Glob. Challenges 3, 1800013. https://doi.org/10.1002/gch2.201800013

Quistberg, D.A., Diez Roux, A. V., Bilal, U., Moore, K., Ortigoza, A., Rodriguez, D.A., Sarmiento, O.L., Frenz, P., Friche, A.A., Caiaffa, W.T., Vives, A., Miranda, J.J., Alazraqui, M., Spinelli, H., Guevel, C., Di Cecco, V., Tisnés, A., Leveau, C., Santoro, A., Herkovits, D., Gouveia, N., Barreto, M., Santos, G., Cardoso, L., de Menezes, M.C., de Pina, M. de F., de Lima Friche, A.A., de Souza Andrade, A.C., Alfaro, T., Córdova, C., Ruiz, P., Fuentes, M., Vergara, A.V., Salazar, A., Cortinez-O’ryan, A., Schmitt, C., Gonzalez, F., Baeza, F., Angelini, F., Dueñas, O.L.S., Higuera, D., González, C., Montes, F., Useche, A.F., Guaje, O., Jaramillo, A.M., Guzmán, L.A., Hessel, P., Lucumi, D., Meisel, J.D., Martinez, E., Kroker-Lobos, M.F., Ramirez-Zea, M., Folger, K.M., Barrientos-Gutierrez, T., Perez-Ferrer, C., Prado-Galbarro, J., de Castro, F., Rojas-Martínez, R., Hernández-Vásquez, A., Diez-Canseco, F., Hammond, R., Dronova, I., Sanchez, B.N., Hovmand, P., Fuchs, R.J., Braslow, J., Siri, J., Auchincloss, A., Langellier, B., Lovasi, G., McClure, L., Michael, Y., Quick, H., Granados, J.T., Garcia-España, F., Stankov, I., 2019. Building a Data Platform for Cross-Country Urban Health Studies: the SALURBAL Study. J. Urban Heal. 96, 311–337. https://doi.org/10.1007/s11524-018-00326-0
